# Spot the new lesion: a case report of intra-MRI cerebral haemorrhage with discussion of previous literature

**DOI:** 10.1093/bjrcr/uaaf046

**Published:** 2025-09-09

**Authors:** Ali Mokhtari, Valeria Onofrj, Carine Neugroschl

**Affiliations:** Post Graduate in Radiology Department, Iris Sud Hospital, Brussels 1050, Belgium; Resident in Radiology Department, CHU Nefissa Hamoud ex-Parnet, Hussein Dey 16005, Algeria; Attending Neuroradiologist, Iris Sud Hospital, Brussels 1050, Belgium; Attending Neuroradiologist, Cliniques Universitaires Saint-Luc, Brussels 1200, Belgium; Neuroradiologist, Iris Sud Hospital, Brussels 1050, Belgium

**Keywords:** brain haemorrhage, physics, emergency, intensive care unit, neuroradiology

## Abstract

The appearance of intracranial haemorrhage on magnetic resonance imaging (MRI) is complex and depends on its evolution over time. The ability of the MRI to detect hyperacute haemorrhage has been debated as it seems to depend on several factors (ie, delay of imaging, MRI field strength, imaging technique). We present the first case of intracranial haemorrhage occurring during MRI acquisition in a 61-year-old female suffering a severe hypoxic-ischaemic encephalopathy from a suicide attempt by hanging. The imaging characteristics of the haematoma differ from what has been described in the past, as it lacks the typical peripheral T2 and T2* hypointensity usually seen in hyperacute haematomas. We discuss previous literature in order to explain these peculiar imaging features.

## Case presentation

We describe the case of a 61-year-old female admitted to the intensive care unit following a cardiorespiratory arrest resulting from a suicide attempt by hanging. The patient’s medical history includes a grade II Ependymoma and a thoracic Melanoma (pT2 N0 M0), both resected, complicated by 2 brain metastases in the left frontoparietal and the right occipital lobes 5 years later, initially managed with radiotherapy (18 Gy) and followed by surgical resection. Of notice, the patient did not have any past medical history of blood hypertension.

On admission, the patient was under a pharmacologic coma with deep sedation and neuromuscular blockade. Initial neurological findings included early initial myoclonus, few facial tremulation and petechias.

## Investigations/imaging findings

Non-contrast brain and cervical CT did not reveal any cerebral haemorrhage or vertebral fractures.

**Figure 1.**
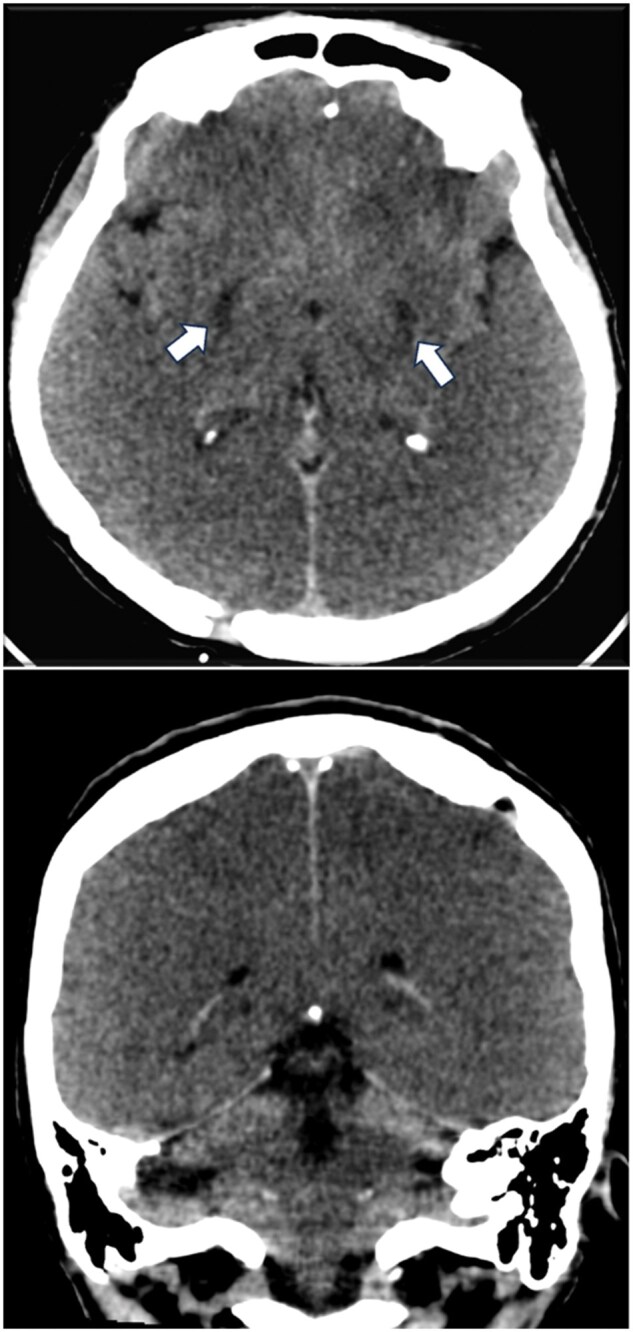
Brain CT demonstrating a diffuse brain swelling with effacement of the sulci, attenuation of grey-white matter interface and a hypodensity in both lenticular nuclei (arrow).

Two days later, after the cessation of sedation, the brainstem reflexes were absent, and the Glasgow Coma Scale score was 5 with decerebrate posturing. Another non-contrast head CT at day 3 was obtained revealing a diffuse brain swelling, effacement of the sulci, attenuation of the gray-white matter interface and a bilateral hypodensity of lenticular nuclei (Figure 1). The CT angiogram was negative.

**Figure 2.**
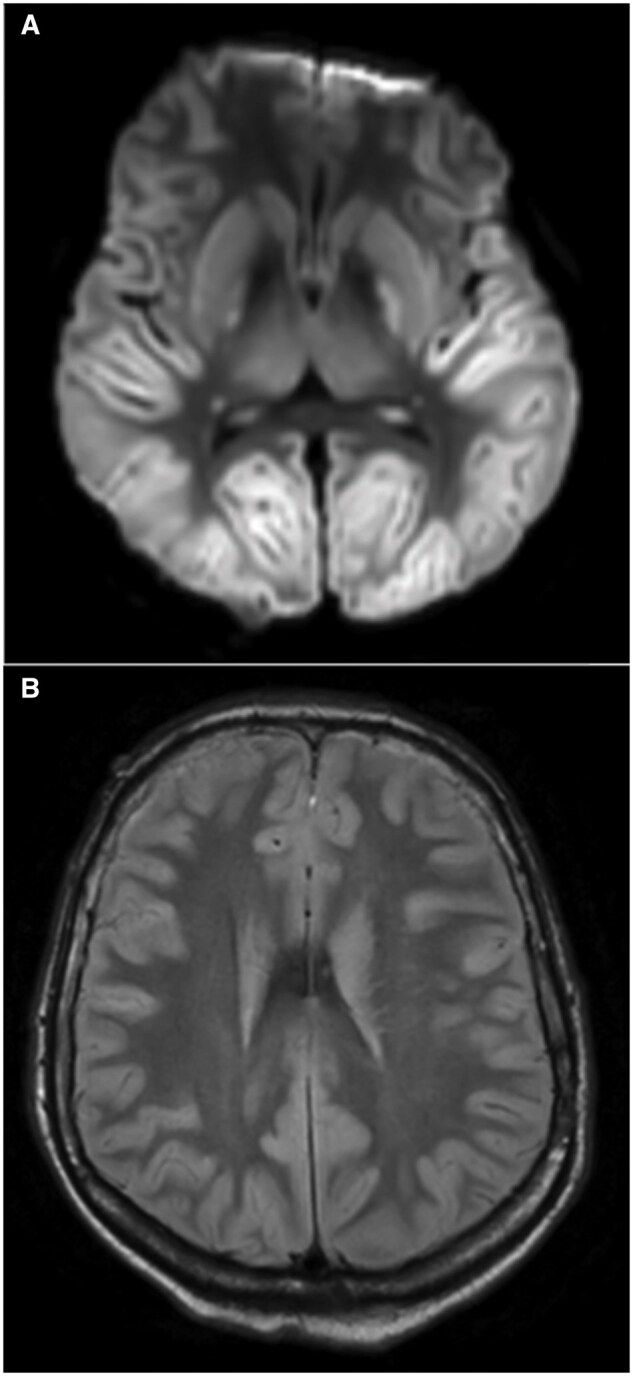
Brain MRI shows diffuse diffusion-weighted imaging (A) and FLAIR (B) hyperintensity in the grey matter and the basal ganglia consistent with a severe hypoxic-ischaemic encephalopathy.

**Figure 3.**
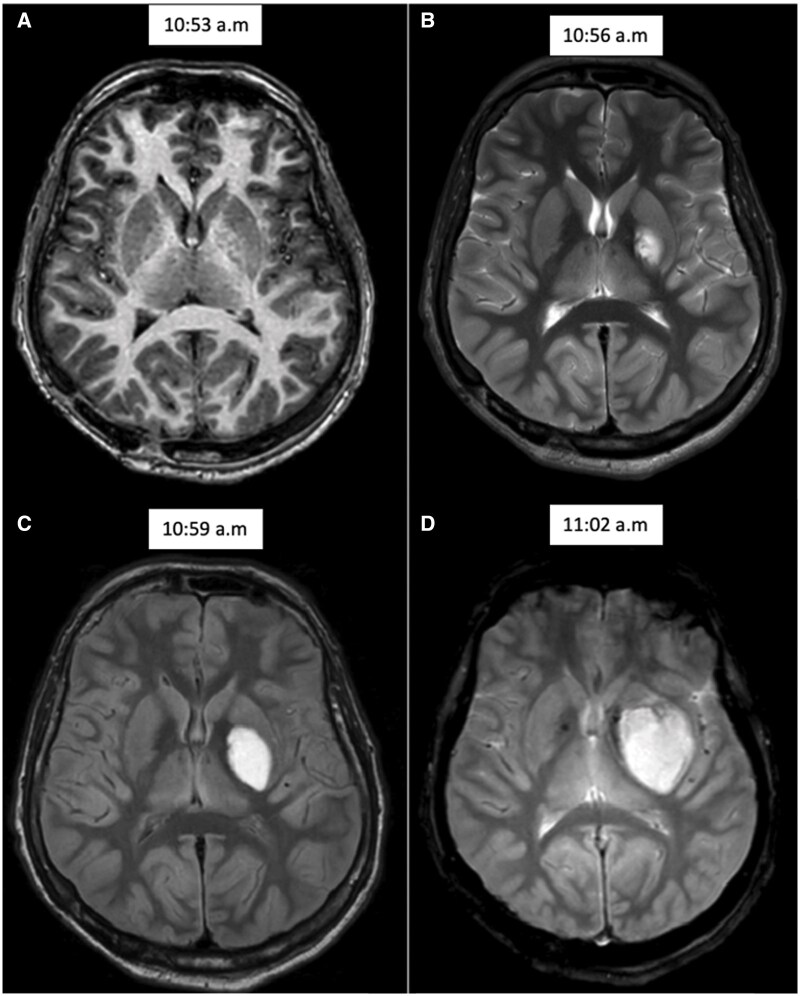
Axial images of the basal ganglia showing a well-defined left globus pallidus collection, hyperintense on FLAIR (C), T2 (B), and T2* (D), increasing during acquisition and non-visible on T1 weighted image (A).

A brain magnetic resonance imaging (MRI) was performed the same day, demonstrating a severe hypoxic-ischaemic encephalopathy on FLAIR and diffusion-weighted imaging (DWI) (Figure 2). It also revealed an atypical finding in the left lenticular nucleus characterized as a well-defined left globus pallidus collection, hyperintense on FLAIR, T2- and T2*-weighted images but not detected on DWI and 3D-T1 sequences (Figure 3).

## Differential diagnosis

Initially, this finding was suspected to be a new metastatic melanotic lesion due to the patient’s oncological history. However, analysis of the MRI sequence chronology indicated that the lesion appeared 3 minutes after the scan began and enlarged progressively during the acquisition. This suggested the diagnosis of an active haemorrhage within the infarcted left lenticular nucleus.

## Treatment

No surgical or medical intervention was performed due to the patient’s severe hypoxic brain injury and rapid clinical deterioration.

## Outcome and follow-up

The patient’s condition markedly deteriorated the following day, which led to the decision to withdraw care, resulting in the patient’s death.

## Discussion

This case report highlights the distinctive imaging features of a hyperacute intracranial haemorrhage captured in real time during MRI acquisition. The lenticular haematoma appeared within 3 minutes of scan initiation, in a patient suffering profound hypoxic-ischaemic encephalopathy after a suicidal attempt by hanging. This unique opportunity to witness the formation of the haematoma in real time provides valuable insight into the MRI appearance of hyperacute intracranial haemorrhage.

**Figure 4.**
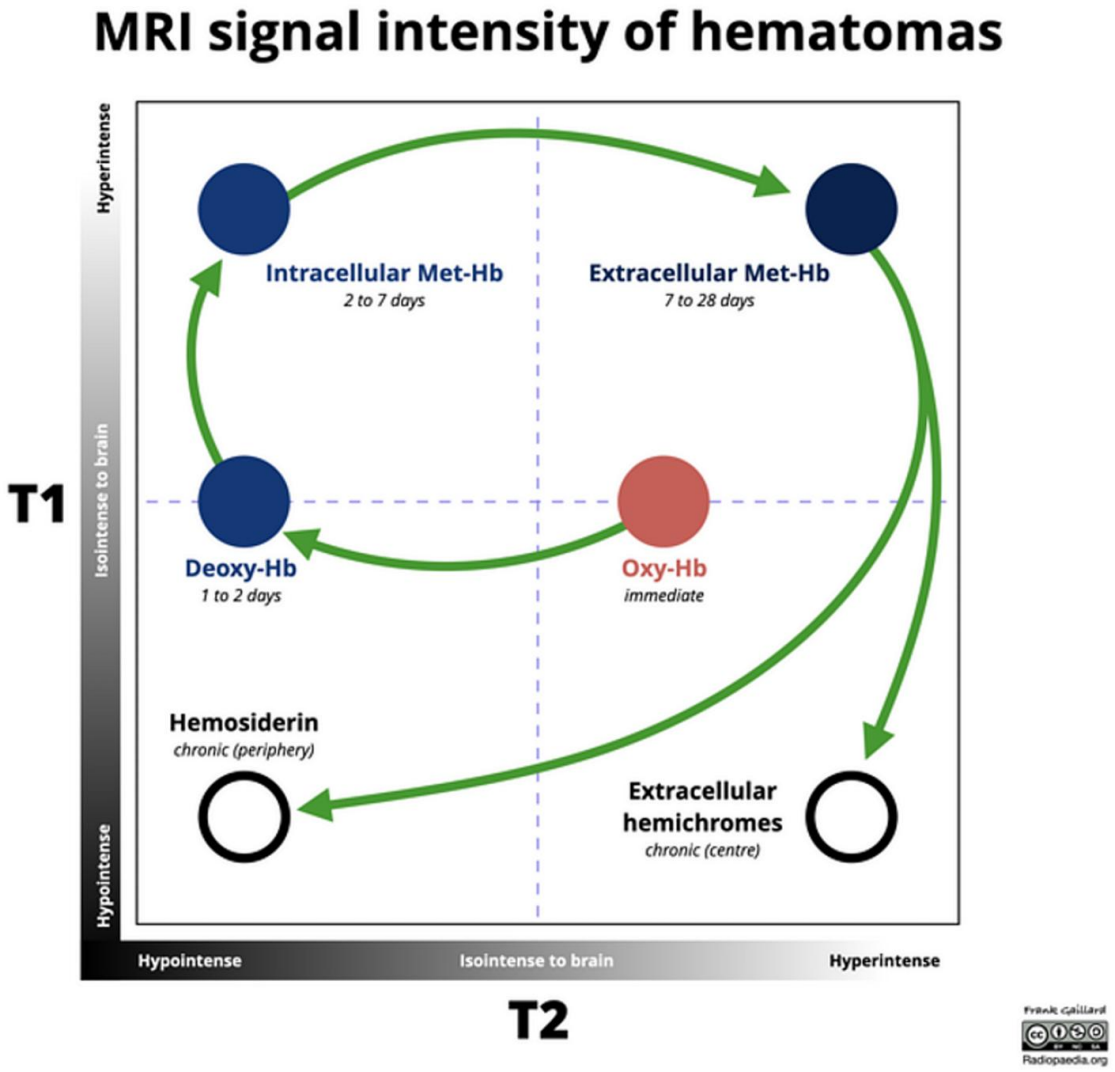
Evolution of MRI signal characteristics of intracerebral haematomas over time.^11^

The MRI appearance of intracranial haemorrhage depends on haematoma age and MR contrast type (T1- or T2-weighted). As a haematoma ages, haemoglobin transitions through oxyhaemoglobin, deoxyhaemoglobin, and methemoglobin before red cell lysis and breakdown into ferritin and hemosiderin (Figure 4). Haemorrhage progresses through 5 stages: hyperacute (intracellular oxyhaemoglobin, long T1/T2), acute (deoxyhaemoglobin, long T1, short T2), early subacute (intracellular methemoglobin, short T1/T2), late subacute (extracellular methemoglobin, short T1, long T2), and chronic (ferritin/hemosiderin, short T2) (Table 1). The short T1 of methemoglobin results from paramagnetic dipole-dipole interactions. Another paramagnetic property, the magnetic susceptibility effect, is responsible for the short T2 observed when deoxyhaemoglobin, methemoglobin, or hemosiderin is intracellular. Additionally, haemoconcentration and clot retraction can contribute to T2 shortening.^1^

**Table 1.** MRI characteristics and evolution of intracerebral haematomas over time.

Stage	Haemoglobin form	MRI appearance (intensity compared to brain)	Age
Hyperacute	Intracellular oxyhaemoglobin	T1: Isointense T2: Isointense to hyperintense T2*/SWI: Hyperintense with thin hypointense rim DWI: High ADC: Low	<1 day
Acute	Intracellular deoxyhaemoglobin	T1: Isointense to hypointense T2: Hypointense T2*/SWI: Marked hypointensity DWI: Low ADC: Low	6 hours-3 days
Early subacute	Intracellular methemoglobin	T1: Hyperintense (T1 shortening) T2: Hypointense T2*/SWI: Persistent hypointensity DWI: Low ADC: Low	3-7 days
Late subacute	Extracellular methemoglobin	T1: Hyperintense T2: Hyperintense T2*/SWI: Hypointensity may persist DWI: High ADC: Low	7-14 days
Chronic	Hemosiderin and ferritin	Periphery: T1: Hypointense T2: Hypointense T2*/SWI: Hypointense rim Center: T1: Iso/hypointense T2: Hyperintense DWI: Low ADC: High SWI: Variable	>14 days

As blood extravasates into tissue, haemoglobin deoxygenates, making deoxyhaemoglobin a paramagnetic substance due to its unpaired electrons. This creates a nonuniform magnetic field, causing rapid proton spin dephasing in T2- and more so in T2*-weighted images. This property of paramagnetic substance is termed magnetic susceptibility effect and results in signal loss best seen in images that are T2- or T2*-weighted.^2^

The chemical properties of heme degradation products make it possible to explain the typical composition of haematoma. In the hyperacute phase (within 6 hours), the haematoma is typically characterized by a central component primarily comprising oxyhaemoglobin distinguished by a hyperintense T2 and T2* signal; the periphery of the haematoma, predominantly comprising deoxyhaemoglobin, is generally hypointense in the T2 and T2* sequences. A rim of T2 and T2* hyperintensity surrounding the haematoma identifies the presence of vasogenic oedema.

In our case, we did not observe the typical T2 and T2* hypointensity in the periphery of the expanding haematoma. An explanation to this finding is that, since the MRI was conducted while the haematoma was forming, the oxyhaemoglobin component of the haematoma was largely dominant over the deoxyhaemoglobin. Since oxyhaemoglobin becomes deoxygenated in a fraction of seconds, it may be that the amount of oxyhaemoglobin exceeds deoxyhaemoglobin because the rate of the haemorrhage expansion exceeds the rate of heme deoxygenation by units of volume. It is possible that if we had repeated the T2* sequence when the haemorrhage stopped expanding, we would have seen the typical peripheral hypointensity.

A major factor in detecting the deoxygenated component of haemorrhage is the magnetic field strength, since it amplifies the proton spin dephasing in T2 and T2* images produced by paramagnetic substances.^3^ This finding partly explains why in previous studies conducted mostly on low-field MRIs, authors stated that MRI is not a sensitive technique to hyperacute haemorrhage.^4^ This statement was corrected in later studies conducted on 1.5T, where it was clear that MRI could detect deoxyhaemoglobin in less than 30 minutes from the onset of symptoms.^2^^,^^4^

Our case report proves that, even at high field strength (3T), the detection of hyperacute haemorrhage on MRI is time dependent. Further studies comparing 3T and 1.5T for haemorrhage detection would provide a better understating of the sensitivity of high field MRI in detecting hyperacute haemorrhage detection.

We hypothesized that the left lenticular haemorrhage was due to haemorrhagic transformation of the hypoxic-ischaemic lesion as a result of reperfusion. In the rare cases of haemorrhagic transformation of hypoxic-ischaemic lesions following cardiac arrest,^5–7^ haemorrhagic transformation occurs symmetrically in both lenticular nuclei or more rarely in the mesencephalic tectum. The monolaterality of haemorrhage in our case could be due to asymmetry of hypoxic ischaemic injury, an eventuality reported in some cases of strangulation,^8^ but less intuitive in a case of hanging. The asymmetry of ischaemic hypoxic injury could have been followed by asymmetric reperfusion injury with unilateral haemorrhage. Since we saw the haemorrhage appear in the left lenticular nucleus during the IRM examination, we speculate that the haemorrhagic transformation in the contralateral lenticular nucleus may have occurred later without being able to be verified. Arterial Spin Labeling (ASL) perfusion imaging could perhaps have identified asymmetric hyperperfusion between the lenticular nuclei.^8^

Another diagnostic hypothesis we had considered, given the known past medical history of brain metastasis from melanoma, was of a secondary haemorrhagic lesion with a haematoma in the making. However, neither the CT nor the first 2 sequences (DWI and 3D-T1) performed before the appearance of the haemorrhage showed an obvious underlying mass, vascular abnormality or any other lesion, which could explain a haemorrhage of such considerable size as in our case.

A limitation of our case is that we did not perform susceptibility-weighted image (SWI) since our imaging protocol was tailored for the detection of hypoxic-ischaemic lesions. SWI is a high-resolution 3D-gradient echo sequence which accentuates the differences between paramagnetic substances (deoxyhaemoglobin, methemoglobin, and ferritin) and brain parenchyma, thereby making this sequence even more sensitive to microhaemorrhage.^9^ SWI has a higher sensitivity compared to T2* techniques for the detection of intracranial blood products.^10^ It is likely that SWI allows earlier detection of deoxyhaemoglobin in hyperacute haemorrhage, although the time limitation in the detection of hyperacute haemorrhage likely depends on the rate of haemorrhage expansion compared to the rate of blood deoxygenation.

## Conclusions

We report the first case of intra-MRI cerebral haemorrhage showing different imaging characteristics compared to typical hyperacute haemorrhage. These findings point to the time limitation of MRI in detecting hyperacute haemorrhage. We also underline that this is the first case reported in the literature of a unilateral lenticular haemorrhage resulting from reperfusion damage of a hypoxic-ischaemic encephalopathy.

## Learning points

- Magnetic resonance imaging detection of hyperacute haemorrhage is time-dependent even if performed at high field strength (3T).
- Hyperacute haemorrhages may not show the classic peripheral hypointensity on T2/T2*.
- Reperfusion haemorrhage should be considered in hypoxic-ischaemic injury, even when unilateral.
- SWI could provide additional sensitivity for early detection of deoxyhaemoglobin compared to T2*sequence, and should be included in relevant protocols.

## References

[1] Bradley WG. MR appearance of hemorrhage in the brain. Radiology. 1993;189:15-26. 10.1148/radiology.189.1.8372185 8372185

[2] Linfante I , Llinas RH, Caplan LR, et al MRI features of intracerebral hemorrhage within 2 hours from symptom onset. Stroke. 1999;30:2263-2267. 10.1161/01.STR.30.11.2263 10548654

[3] Iftikhar S , Rossi N, Goyal N, et al Magnetic resonance imaging characteristics of hyperacute intracerebral hemorrhage. J Vasc Interv Neurol. 2016;9:10-13. 27829965 PMC5094255

[4] Patel MR , Edelman RR, Warach S. Detection of hyperacute primary intraparenchymal hemorrhage by magnetic resonance imaging. Stroke. 1996;27:2321-2324. 10.1161/01.str.27.12.2321 8969800

[5] Boukobza M , Baud FJ. Hemorrhagic infarct of basal ganglia in cardiac arrest. CT and MRI findings. 2 cases. Neurol Neurochir Pol. 2018;52:94-97. 10.1016/j.pjnns.2017.09.004 28965668

[6] Finelli PF. Bilateral hemorrhagic infarction of the pallidum. J Comput Assist Tomogr. 1984;8:125-127. 10.1097/00004728-198402000-00025 6690494

[7] Gilles FH. Hypotensive brain stem necrosis. Selective symmetrical necrosis of tegmental neuronal aggregates following cardiac arrest. Arch Pathol. 1969;88:32-41. 5793687

[8] Prosser DD , Grigsby T, Pollock JM. Unilateral anoxic brain injury secondary to strangulation identified on conventional and arterial spin-labeled perfusion imaging. Radiol Case Rep. 2018;13:563-567. 10.1016/j.radcr.2018.02.004 29988732 PMC6030549

[9] Jain N , Kumar S, Singh A, et al Blood in the brain on susceptibility-weighted imaging. Indian J Radiol Imaging. 2022;33:89-97. 10.1055/s-0042-1758880 36855723 PMC9968548

[10] Shams S , Martola J, Cavallin L, et al SWI or T2*: Which MRI sequence to use in the detection of cerebral microbleeds? The Karolinska Imaging Dementia Study. AJNR Am J Neuroradiol. 2015;36:1089-1095. 10.3174/ajnr.A4248 25698623 PMC8013035

[11] Gaillard F. Evolution of MRI signal characteristics of intracranial hemorrhage (diagram). Case study, Radiopaedia.org. Accessed September 10 2025. 10.53347/rID-36065

